# A Scalable and Low Stress Post-CMOS Processing Technique for Implantable Microsensors

**DOI:** 10.3390/mi11100925

**Published:** 2020-10-05

**Authors:** Ah-Hyoung Lee, Jihun Lee, Farah Laiwalla, Vincent Leung, Jiannan Huang, Arto Nurmikko, Yoon-Kyu Song

**Affiliations:** 1 Graduate School of Convergence Science and Technology, Seoul National University, Seoul 08826, Korea; ahhyoung@snu.ac.kr; 2 School of Engineering, Brown University, Providence, RI 02912, USA; jihun_lee@brown.edu (J.L.); Farah_Laiwalla@brown.edu (F.L.); 3 Department of Electrical and Computer Engineering, Baylor University, Waco, TX 76706, USA; vincent_leung@baylor.edu; 4 Department of Electrical Engineering, University of California, San Diego, CA 92161, USA; jih324@eng.ucsd.edu

**Keywords:** complementary metal–oxide–semiconductor (CMOS) post-processing, polydimethylsiloxane (PDMS)-assisted planarization, on-chip electrode, scalable chip integration technique, BMI (brain–machine interfaces), wireless, biomedical implants

## Abstract

Implantable active electronic microchips are being developed as multinode in-body sensors and actuators. There is a need to develop high throughput microfabrication techniques applicable to complementary metal–oxide–semiconductor (CMOS)-based silicon electronics in order to process bare dies from a foundry to physiologically compatible implant ensembles. Post-processing of a miniature CMOS chip by usual methods is challenging as the typically sub-mm size small dies are hard to handle and not readily compatible with the standard microfabrication, e.g., photolithography. Here, we present a soft material-based, low chemical and mechanical stress, scalable microchip post-CMOS processing method that enables photolithography and electron-beam deposition on hundreds of micrometers scale dies. The technique builds on the use of a polydimethylsiloxane (PDMS) carrier substrate, in which the CMOS chips were embedded and precisely aligned, thereby enabling batch post-processing without complication from additional micromachining or chip treatments. We have demonstrated our technique with 650 μm × 650 μm and 280 μm × 280 μm chips, designed for electrophysiological neural recording and microstimulation implants by monolithic integration of patterned gold and PEDOT:PSS electrodes on the chips and assessed their electrical properties. The functionality of the post-processed chips was verified in saline, and ex vivo experiments using wireless power and data link, to demonstrate the recording and stimulation performance of the microscale electrode interfaces.

## 1. Introduction

With sustained innovation over decades, the opportunities offered by complementary metal–oxide–semiconductor (CMOS) microelectronics have transformed the electronics of active biomedical implants, taking advantage of the low power consumption, low-cost scalability, and a variety of electronic functions (digital/analog signal processing, data memory, storage, etc.), including systems-on-chip. Examples range from MEMS (microelectromechanical) integrated systems [1,2] to microfluidic devices [3,4,5,6], and bio/chemical sensors [7,8,9,10,11]. Among those applications, the development of wireless microscale neural implants using CMOS has been explored as one approach for next-generation brain–machine interfaces (BMI) by several groups [12,13,14,15,16,17,18]. Here, each untethered implantable microdevice is its own system-on-chip, shrinking conventional neural electronics to sub-mm scale, typically combining analog and digital circuits with radiofrequency (RF) or equivalent wireless telemetry functions.

Once a bare silicon die arrives from a semiconductor fab, a process challenge must be met to enable subsequent post-CMOS processing and packaging steps to complete the functional implant. These might include building biocompatible microelectrodes on contact pads, applying coatings for hermetic sealing, and so on, which typically requires microfabrication processes such as lithography, metallization, etching, and bonding on the CMOS die. In a CMOS process, aluminum that is often used as a top metal is oxidized over time, which can disrupt the electrical interface with biological tissue, as one example [8,9]. For this problem, stable and biocompatible alternative materials such as gold (Au), platinum–iridium alloy, and poly(3,4-ethylenedioxythiophene) polystyrene sulfonate (PEDOT:PSS) are attractive to provide long-term reliability of the electrode–tissue interface and electrode–electrolyte impedance optimization [19,20,21,22,23,24]. One post-CMOS strategy has made use of the sacrificial carrier substrate accompanying the bare die that holds the individual microdevices, using deep-etched silicon, silicon oxide substrate [25,26,27], or rigid polymer [5,6,28,29,30]. However, in the case of using the silicon-based carrier substrate, the size variance of chips, generated during a dicing process, can result in discontinuity on the surface of chip-to-carrier assembly. Furthermore, the rigid polymers used in previous studies, such as SU-8 photoresist or epoxy resin chosen for their high chemical resistance, often do not provide a simple way to retrieve the chips after post-processing. In particular, as one moves in the future towards ever smaller chips such as the 22-nm transistor node and below [31,32], the smaller die or contact pad size is likely to affect the process yield as finished chips are fragile and difficult to handle, especially when the silicon chip is thinned to around 30 μm, making any post-CMOS process extremely challenging.

To overcome such hurdles, and described in detail below, we have developed a novel chip processing technique using a polydimethylsiloxane (PDMS)-chip assembly approach, and applied this as a post-CMOS process-flow on ensembles of diced and thinned semiconductor chips. The use of PDMS of appropriate compliance allows embedding of bare CMOS chips into the soft polymer in the form of a 2D grid and in a well-aligned planar geometry without complicated micromachining to fabricate the sacrificial carrier and additional processes for leveling and filling. By forming this planar geometry, our process allows the application of the common microfabrication technique on sub-mm chips in a reversible manner, allowing chip retrieval, which has not been demonstrated previously to our knowledge. In addition, the entire fabrication is designed as a dry assembly process that does not involve harsh chemicals, mechanical stress, and high temperatures to prevent the damage on the CMOS device. Using this type of PDMS-assisted planarization method, we could firstly print pairs of Au and PEDOT:PSS microelectrodes with several different sizes and shapes directly on the contact pads of the ultra-miniature chips (280 μm and 650 μm in area, and 30 μm in thickness) through photolithography and electron beam deposition under high-resolution control. As another key step in the post-process flow, we applied laser ablation to write individual wireless addresses on ensembles of chips for their wireless network telecommunication. Finally, we have conducted benchtop tests in saline and an ex vivo experiment to verify the recording and stimulation performance of the fully processed inductively powered microdevices.

## 2. Materials and Methods

### 2.1. Planarized 2D Chip Array

For the alignment of multiple chips as an array, an important first step to enable further device processing, markers were created on a glass slide by photolithography. The glass substrate was cleaned with acetone, IPA, and DI water and dried with nitrogen immediately. An adhesion promoter (hexamethyldisilazane, HMDS) was first spin-coated on the glass at 2500 rpm for 15 s, a thin photoresist layer (AZnLOF2035, MicroChemicals GmbH, Ulm, Germany) was spun at 3000 rpm for 60 s, then was soft baked on a hotplate at 100 °C for 90 s, and exposed to 80 mJ/cm² UV light at 375 nm. After post-baking (same condition as the soft-bake), the photoresist was developed in AZ MIF-300 for 10 s. The predesigned photomask patterns, including a set of L-shaped alignment marks at the four corners of each chip and pads that we have implemented on the chips, were precisely transferred on the glass to create an alignment template to define the target location map for chips to come. Then, a homogenous mixture of PDMS and a curing agent (Sylgard 184, Dow Corning, Midland, MI, USA), at a ratio of 10:1, was directly spin-coated on the glass substrate with photoresist markers at 3000 rpm for 60 s and cured for 7 min at 90 °C. The thin sacrificial PDMS layer served to protect photoresist markers from damage and provided an adhesive surface for the chips.

**Figure 1:**
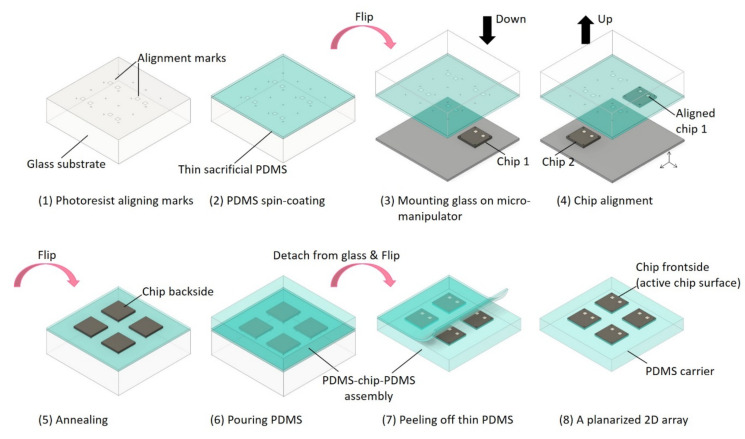
The polydimethylsiloxane (PDMS)-based fabrication procedures for 2D-planarized chip arrays for post-processing: (**1**) A patterned array of photoresist aligning marks is created on a glass substrate. (**2**) The glass substrate is spin-coated with the first 65 μm-thick PDMS layer. (**3**) After baking, the glass substrate is mounted on a manipulator with the adhesive PDMS layer facing down. (**4**) The chips are aligned and picked up by the sticky PDMS surface, guided by the markers. (**5**) The glass substrate with the chips is detached from the manipulator and annealed. (**6**) With the glass substrate facing down, the second layer of planarizing PDMS is poured over the chips and cured overnight. (**7**) After detaching the glass substrate, the spin-coated PDMS layer is carefully peeled away. (**8**) The complementary metal–oxide–semiconductor (CMOS) chip array with active circuits exposed and flushed with PDMS carrier is ready for post-process fabrication.

Subsequently, the PDMS-coated glass carrier was mounted upside down on a micromanipulator under an optical microscope, facing a given CMOS chip mounted loosely on a bottom stage. While microcontrolling the position of the optically transparent marker in the xy-plane, the glass carrier was lowered to the point of a gentle touch to pick up the CMOS chip with the active circuits in contact with the adhesive PDMS surface. On average, 3–4 chips can be aligned within 1 min. By repeating this step, multiple chips could be aligned precisely as a 2D array. Following the pick-and-place procedure, the glass substrate with an ensemble of precisely positioned chips was detached from the manipulator and annealed at 130 °C on the hot plate for 1 h to prevent adhesion of a second PDMS layer which was deposited next for the purpose of planarizing the chip array. Here, the PDMS mixture was poured judiciously over the glass/PDMS/chip construct to a thickness required for the backside of the chips becoming visibly submerged. The preparation was then cured overnight at 80 °C (> 8 h). In general, this step can be adjusted to match the die thickness of a given set of CMOS chips. After curing, the PDMS-chip-PDMS assembly was detached from the glass and attached to a silicon wafer substrate with the active chip surface facing upward, using double-sided Kapton tape. Finally, the thin sacrificial PDMS layer was gently peeled off from the second PDMS layer, in which the chips now remained embedded as a planarized 2D array. Note that the multistep process eliminated the use of harsh chemicals, excessive mechanical force/pressure, or heat/stress to ensure that the functional CMOS circuits were not damaged. As an added benefit, the PDMS-chip assembly could be disassembled if needed, without leaving any polymer residue on the CMOS active chip surface after the full cycle of post-processing steps was completed. Figure 1 graphically elaborates the PDMS-based fabrication procedures for 2D-planarized chip array.

### 2.2. Fabrication of On-Chip Microelectrodes with Au and PEDOT:PSS

As the first application example, we describe the post-process fabrication of microelectrodes on a sub-mm sized CMOS die. The on-board circuits were designed for electrophysiological sensing or actuation by wirelessly networked ensembles of implantable microdevices, based on Taiwan semiconductor manufacturing company (TSMC) 65 nm RF low power (LP) and MagnaChip/SK Hynix 180 nm CMOS processes, respectively. Depending on their function, individual chips of either 650 μm × 650 μm or 280 μm × 280 μm area were diced and some of the dies were then mechanically ground to have a resultant ~30 μm thickness by the company (DISCO Hi-TEC, Europe). Each chip was configured for two or three 65 × 65 μm^2^-sized aluminum (Al) pads, commonly available in the CMOS process, to provide direct electrical access to adjacent biological tissue. The impedance and longevity of implanted electrodes in the tissue call for careful consideration of the electrode material. In this work, we employed combinations of Au and PEDOT:PSS thin films as planar microelectrodes, coated on the Al-pads of the CMOS die to complete the contact structure. Unless otherwise specified, all contacts were approximately (70 µm)^2^ in area. For photolithography, we used both a mask aligner (Karl Suss MJB3, Karl Suss America, Waterbury, VT, USA) and a maskless aligner (MLA150, Heidelberg Instruments, Heidelberg, Germany).

The PDMS-planarized chips (Section 2.1) were first plasma-treated in a Fischione model 1070 plasma cleaning system in a 30 sccm of 25% O_2_ and 75% N_2_ gas mixture for 2 min. With the resulting hydrophilic surface modification on the PDMS, this process improves the bonding and printability of the subsequent photoresist and helps one to achieve an even coating over the surface of PDMS-chip assembly. Immediately after plasma treatment, AZnLOF2035 photoresist was first spin-coated on the PDMS-chip surface at 3000 rpm for 60 s and soft-baked in the 92–94 °C oven for 30 min, then exposed with a wavelength of 365 nm at an irradiance intensity of 75 mJ/cm². After baking, patterns defining the desired electrode openings were developed in AZ MIF-300 solution. An electron beam evaporator (Lesker Lab-18, Kurt J. Lesker Company, Jefferson Hills, PA, USA) was used for the deposition of Ti/Au (50/200 nm) thin films followed by the lift-off process using AZ 400T stripper on the 80 °C hot plate for 5 min. As for adding the conductive polymer thin film coating, 20 mL aqueous dispersion of PEDOT:PSS (Clevios PH1000, Heraeus Epurio Clevios, Hanau, Germany) was mixed with 5 mL ethylene glycol, 50 μL dodecylbenzene sulfonic acid (DBSA), and 1 wt% of (3-Glycidyloxypropyl) trimethoxysilane (GOPS). Adding ethylene glycol enhances the conductivity of PEDOT:PSS, while DBSA helps to increase its coating properties by adjusting the surface tension. In addition, GOPS enhances the stability of PEDOT:PSS films in aqueous environments as a cross-linker [24]. Before applying PEDOT:PSS film on the Au/Al contact pads, AZ photoresist was spin-coated and exposed to UV aligning the PEDOT:PSS electrode sites in co-registry with the CMOS chip contact pads. The mixture of PEDOT:PSS was then evenly spin-coated onto the 3.5 μm-thick AZ-photoresist-patterned chips at 3000 rpm for 60 s and soft-baked in the 130 °C oven for 20 min. The AZ photoresist was lifted off in all regions except PEDOT:PSS electrode sites using acetone under sonication for 1 min. Note that since PEDOT:PSS films are susceptible to a base solution, acetone was only used to dissolve AZ photoresist patterns in the lift-off process [33,34]. The PEDOT:PSS was subsequently cured on the 130 °C hot plate for 1 h.

### 2.3. Characterization of Microelectrode Electrical Impedance

Since PEDOT:PSS offers biocompatible interfaces between electrode and biological tissue and improves the efficiency of stimulation current delivery due to its low contact impedance, it has emerged as one of the promising electrically conductive polymers to be used as microelectrode material [19,20,21,22,23,24]. For our impedance measurement, we fabricated 70 μm square Au and PEDOT:PSS-coated Au electrodes with extended structures, including additional pads on the chip surface. This chip was attached to a flexible liquid crystal polymer (LCP) film with patterned Au traces. Using a ball bonder outfitted with a 20 μm diameter Au bond wire (WestBond, USA), two extended Au pads on the opposite electrodes were wire-bonded on the LCP substrate individually, which helped to make the external connections with the tester during measurement. This structure is encapsulated using an epoxy resin except for the Au and PEDOT:PSS electrode sites exposed to saline solution. Among two exposed on-chip electrodes, one serves as the active electrode while the other serves as the reference electrode. The impedance measurement is performed at room temperature and the frequency sweep is from 1 Hz to 2.04 kHz using a NanoZ impedance tester (White Matter LLC, Seattle, WA, USA).

### 2.4. Post-Processing on-Chip Device Identifiers by Laser Ablation

Wireless microimplants often require unique RF device identification. The 2D planar array microfabrication platform technique presented here is also compatible with the standard CMOS post-processing technique, such as laser ablation to write addresses on individual chips. Here, 10-bit metal fuses were cut selectively to assign the different binary bits addresses to each chip using a commercial EzLaze 3 laser cutting system (New Wave Research, Sunnyvale, CA, USA) with the manual operation. Pulsed 355 nm UV laser radiation was first utilized to ablate a window in the dielectric passivation (scratch protect) bilayer (Si3N4/SiO2) on top of the chip’s surface to access the Al metallization features in the designated RF address bus embedded within the CMOS circuit. Considering the fuse size, the laser target spot size for window opening was set to 2.5 μm × 15 μm defined using a 50 objective (7–8 single shot exposures were sufficient to remove the dielectric layer). Subsequently, 532 nm green laser light was used to cut the Al-metal traces within the fuse. We chose a smaller aperture size (2.5 μm × 10 μm) to reduce physical damage on the proximate dielectric layer (3–4 times exposures single shot exposures). The laser-ablated fuse address was registered by each chip’s internal circuitry and validated electronically through wireless radiofrequency (RF) power and data transmission. In brief and while described in more detail elsewhere, wireless communication deployed an on-chip receiver microantenna (Rx) coil and inductively coupled to an external coil (Tx) resonant at 915 MHz. An RF backscatter modulation circuit on the chip produced a binary phase-shift keying (BPSK)-encoded signal, which was recovered by an external RF receiver. The laser-written 10-bit address and an interleaved header sequence were extracted from the backscattering signal after demodulation [35].

### 2.5. Assessing the Functionality of Post-Processed Microchips

The active microchips were designed for eventual use as distributed implants, codenamed “neurograins” in our laboratory [12,13,35,36,37,38,39]. After the post-processing steps, the recording performance of the neurograin chips with on-chip Au planar microelectrodes was first demonstrated in a saline immersion benchtop test under RF wireless powering [13]. A concentric platinum–iridium oxide electrode was used to inject 200 Hz sinusoidal proxy neural signal into the saline bath in which the chips were immersed, through an arbitrary waveform generator. The signals were recorded by the chips and information backscattered to the external antenna and electronics. In another series of experiments, physiological validations of the microstimulation performance of the neurograin using PEDOT:PSS coated Au electrodes were assessed both in saline and GFAP Cre × ChR2-YFP mouse brain slices. In the brain slice experiments, a 200 μm-thick coronal slice was immersed in a fluid chamber which was continuously perfused with artificial cerebrospinal fluid (ACSF). The wireless telemetry components were fully integrated within the chamber. A stimulating neurograin was positioned on the cortical tissue and standard extracellular recording techniques were used to monitor the stimulation-evoked neural activity. A 100 Hz, 25 μA symmetric stimulation pulse train is injected by the neurograin through the PEDOT:PSS electrodes into the brain slice to evoke local field potential responses. 

## 3. Results and Discussion

In this section, we present the results from the application of the methods (Section 2), while expanding and elucidating these further for specific process-critical points of the CMOS chip post-fabrication. 

### 3.1. The Planarized Fabrication Process with a 30 μm Thin Die

**Figure 2:**
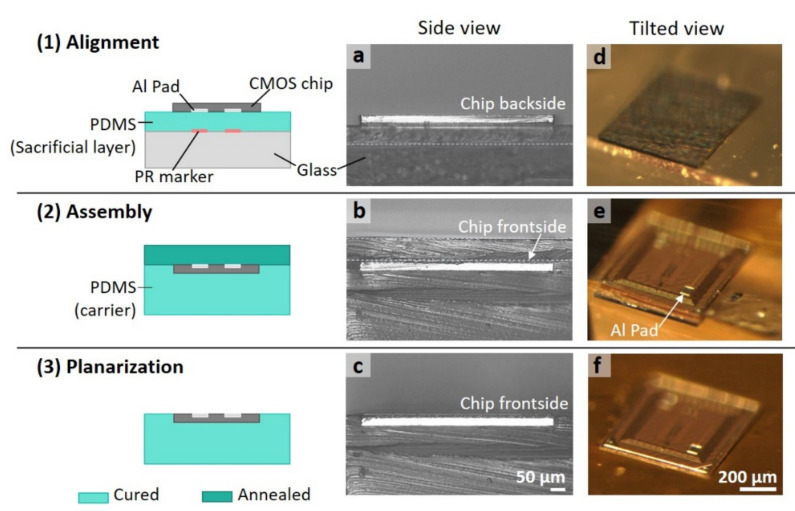
Summary of the alignment, assembly, and planarization processes of a sub-mm sized CMOS chip (left column shows steps (**1**)–(**3**)), next to microscope optical images in side view (a–c) and tilted view (d–f) for each step in the 2D planarization process, here using a thinned CMOS chip (650 μm × 650 μm × 30 μm): Step (**1**) The chip facing down lands on the spin-coated PDMS film (65 μm thickness) aligned with its corresponding photoresist (PR) marker. Step (**2**) The chip is sandwiched between two PDMS layers (after annealing). Step (**3**) The final planarized chip/PDMS assembly is completed after the top PDMS layer is peeled off from the contacting chip/PDMS surface. Note the two small square Al-contact pads on the chip discussed below and in text.

The 2D PDMS-carrier planarization strategy was implemented on both commonly used 200–300 μm thick and 30 μm thin silicon CMOS chips, to highlight the adaptability of this method to the die thickness. Figure 2 complements Figure 1 above by showing photographic snapshots from the PDMS layering process steps during the planarization of a foundry-produced sub-mm scale CMOS chip, here 650 μm × 650 μm × 30 μm in size. Cross-sectional images showing the chip being sandwiched by PDMS layers are seen in the side view (a–c) and the angled view (d–f), respectively. Optical micrographs provided a detailed estimation of the two-dimensional layer structure and the alignment precision. Notably, the first PDMS layer with 65 μm thickness adheres to the chip tightly enough to prevent the second PDMS from smearing underneath the chip during the formation of the assembly. A successful planarized chip alignment was defined by a visibly smooth, uniform, void-free, and expanded surface observed under the microscope. By mounting the finalized PDMS-chip assembly on a silicon wafer carrier, the subsequent microfabrication process could commence. An advantage of our approach is that the final planarized chip/PDMS assembly (step 3) kept its initial form without distortion and degradation during post-processing due to the chemical stability and mechanical properties of PDMS, including its moderate stiffness and surface adhesion energy that held the assembly together.

### 3.2. Surface Profile of the Photoresist Coated Sub-mm Sized Single Die

**Figure 3:**
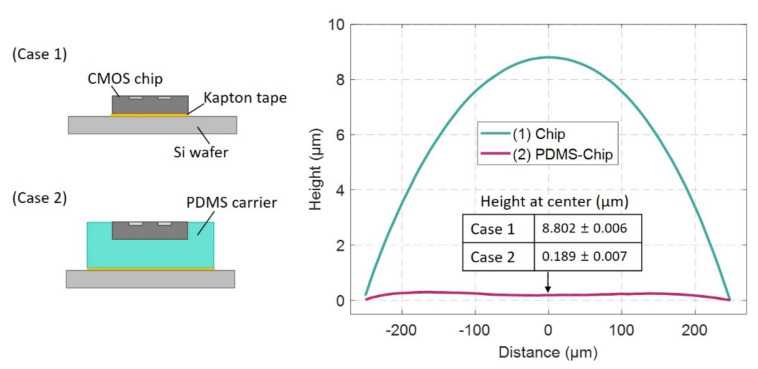
Comparing the surface profiles microchips coated by a 3.5 μm-thick photoresist film, without (case 1) and with the planarization process (case 2). Both samples were spin-coated at 3000 rpm for 60 s and baked in the oven at 90 °C for 30 min. The unprocessed and PDMS-planarized chips (650 μm × 650 μm × 200 μm) were attached on the silicon wafer using a double-side Kapton tape.

To test the surface smoothness of the planarized chip/PDMS arrays, we compared the surface profiles of a single chip with and without the planarization step by spin-coating a reference photoresist layer on each (photoresist being a key tool in subsequent post-processing). A 3.5 μm-thick photoresist layer was spin-coated on both samples attached to a silicon wafer as a carrier, and the uniformity of the photoresist was measured with a profilometer (Dektek 3, Veeco Instrument, Plainview, NY, USA) by scanning a distance of 500 μm at a speed of 0.25 μm/s. Figure 3 shows the average profile of five consecutive measured values at the same position. Note how for the case of no PDMS-based planarization (case 1), the spin-coated photoresist only resulted in a curved droplet profile on the chip surface. The photoresist height increased toward the center of the chip and peaked at the midpoint value, 8.802 ± 0.006 μm. This is understandable as the surface tension of the photoresist on a small area surface is dominant in comparison to the centrifugal force by the spinner. By contrast, the planarized chip with PDMS shows much better uniformity and coverage of the photoresist film with only 0.189 ± 0.007 μm roughness across the same area (case 2). The result suggests that the PDMS-assisted planarization process effectively extends the spin-coating boundaries to create a uniform sheet of surface coverage by balancing the forces acting on the photoresist, an outcome which is necessary for the subsequent post-processing steps. 

### 3.3. High Precision Post-Process Micropatterning of Microelectrodes on Chip Ensembles 

**Figure 4:**
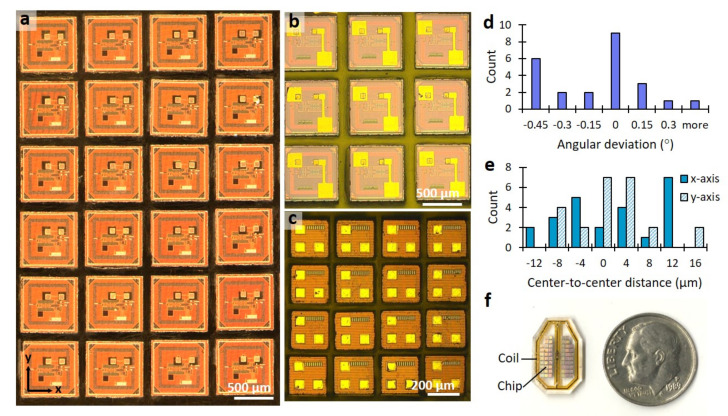
Examples of post-processing multiple CMOS chips through batch fabrication: (**a,b**) the array of 650 μm × 650 μm chips with 100 μm or 200 μm square Au patterns on two Al-pads within each chip; (**c**) the array of 280 μm × 280 μm chips with 70 μm square Au patterns on three Al-pads within each chip; (**d,e**) histogram of the measured angular orientation and center-to-center lateral distance; (**f**) size comparison with a dime and arrays of 56 chips within a polyimide RF antenna coil.

A key goal of this work was to develop a method to enable high throughput batch post-processing of multiple sub-mm scale CMOS dies. Figure 4 demonstrates how the method is indeed scalable toward a batch fabrication process, including CMOS chips of different footprints. Figure 4a shows an array of 650 μm × 650 μm × 200 μm sized chips, where a pair of 100 μm sized square Au microelectrode patterns are deposited on corresponding Al contact pads (as received from the fab). In Figure 4b, 200 μm square Au patterns could be added around the proximate seal ring structure surrounding the edge side of the chip. Figure 4c shows a high precision example of miniature chips as small as 280 μm × 280 μm × 200 μm having been successfully patterned by square Au dots on Al contact pads. To quantify our alignment accuracy, the angular deviation and center-to-center distance were measured for the array of Figure 4a by overlaying the photomask CAD drawing. During the calculation, the alignment offset of the mask image was canceled by subtracting the average of the measured values. The CAD measurement guide includes a grid of squares on pad sites and alignment marks at the four corners of each chip with a regular pitch and angular orientation. The result of the measured angular deviation is shown in Figure 4d. The achieved x–y precision is shown in Figure 4e by measuring the center-to-center distance between the center of two pads on the CAD drawing and that of actual chips. The accuracy of the process was determined using the standard deviation, which was 0.3° for the angular orientation, and 8.5 μm and 6.4 μm for the lateral accuracy on the x-axis and y-axis, respectively. The results show the alignment accuracy is well within the range of tolerance required for our implantable microchip fabrication processing. An example of a real multichip sensor array is shown in Figure 4f, discussed below. If it were necessary to further improve the accuracy and scalability of our technique to thousands of chips, we envision an automated alignment procedure for large ensembles using a robotic microhandling tool guided by a computer vision system. This advancement would also improve the process yield by reducing the number of misaligned chips and the fabrication time required for chip placement.

**Figure 5:**
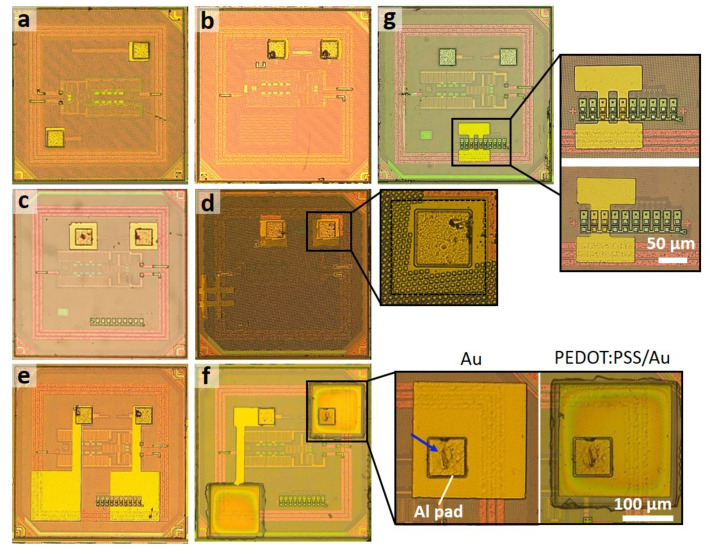
Examples of post-processed Au-micropatterns on chips with different electrode sizes and layouts: (**a,b**) pair of 70 μm square pads, (**c,d**) pair of 100 μm square pads, and (**e,f**) the extended electrode contact pad patterns; (**g**) The post-processed die with an on-chip fuse structure demonstrating high-resolution alignment control (dimension of 12 μm × 35 μm for individual fuse and 5 μm spacing). The insets show a close-up view of micropatterns with both Au and PEDOT:PSS coating on targeted planar microelectrode sites. The blue arrow shows the indentation generated by the mechanical removal of the Al oxide layer.

**Figure 6:**
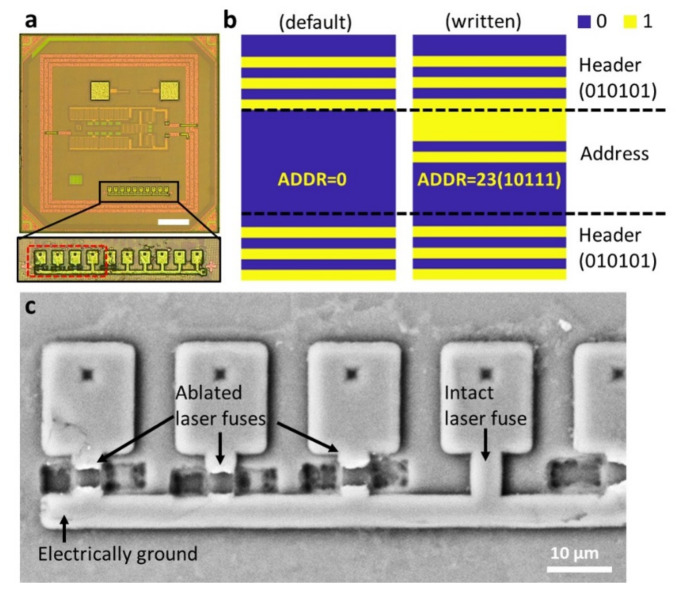
(**a**) A microscope image of a CMOS chip with 500 μm × 500 μm sized RF on-chip antenna coil and a close-up view of an embedded 10-bit fuse structure. Scale bar: 100 μm. (**b**) Validation of addressing from RF backscattered data for the 10-bit address and an interleaved header sequence (010101) [35]. Default code is 10b’0; the laser-written address sets a distinctive identity (10111 = 23 in this case). (**c**) SEM image of the one fuse structure after the laser ablation process.

We also tested the ability of the method to accommodate different layouts and contact electrode sizes in a batch process (Figure 5). Figure 5a,b illustrate the case of 65 μm × 65 μm-sized Al-pads in different locations on chips which were targeted with the deposition of 70 μm square Au thin films. In another case, Figure 5c,d show the results of depositing 100 μm square Au patterns on a chip without or with metal dummy fills (choice in the CMOS process), with different center-to-center spacing of 120 μm and 92.5 μm, respectively. More complex layouts were also possible, as shown in Figure 5e,f, where the electrical connection between Al-pads and 180 μm square pads is fabricated for a larger surface area and electrode separations. The structure in Figure 5e was used to measure the impedance of Au and PEDOT:PSS electrodes, while the overall pad structures provided electrical access to multiple sites, as described in Section 3.5. Additionally, Figure 5g includes two Au fuse structures embedded in the CMOS chips, illustrating an alignment control in our method at the resolution of a few micrometers. The fuse structure has dimensions of 12 μm × 35 μm with 5 μm unit spacing and serves as an RFID address (radiofrequency identification), discussed in the text and shown further below in Figure 6.

### 3.4. On-Chip Device Identifier for RF Telecommunication

As outlined in Section 2.4, another post-process step of importance to our work in the development of distributed neural microimplants is to enable telecommunication under RF wireless powering. Individual CMOS chips require a unique device address, which we have chosen to implement as a 10-bit fuse structure using a 1.4 μm-thick Al metal micropattern added to the top layer redistribution metal available in the 65 nm CMOS process (Figure 6a). In our design, ten metal lines of the 10-bit fuse structure are electrically connected to the ground within the internal circuitry underneath the dielectric passivation layer. After cutting four of the ten fuses selectively through the laser ablation process (Section 2.4), a specific array of ablated and intact laser fuses was validated by scanning electron microscopy (Figure 6c). In the design, to conserve valuable silicon real estate, each fuse line was constrained to a 3 μm width and 6 μm length. The wireless RF link recognized the laser-written 10-bit individual addresses via the RF backscattered uplink. By demodulating this RF signal, the success in the post-processing of the laser-ablated address was validated electronically as in Figure 6b. The device ID is used for downlink communication. When the transmitter sends the downlink code, the chip decodes the downlink and generates current stimulation only if the downlink code matches the device ID. By using this method, stimulation has been delivered to the brain slice selectively among multiple stimulation neurograins (see performance validation in Section 3.6). The RFID address writing technique is compatible with planarization multichip arrays and semi-automatic machines if large-scale ensembles are required.

### 3.5. Impedance Measurement of On-Chip Microelectrodes

**Figure 7:**
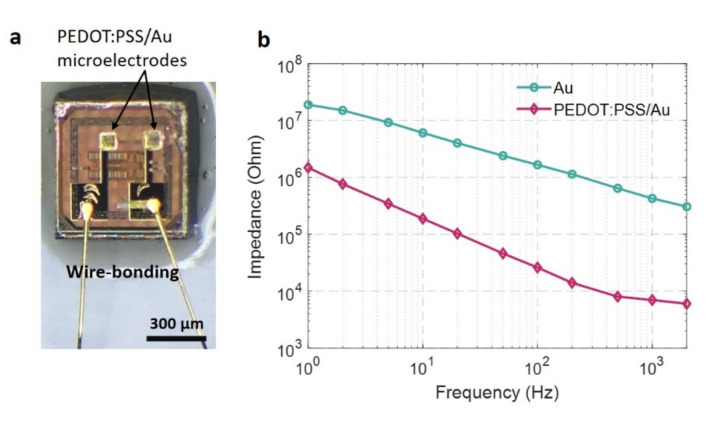
(**a**) An optical image of 70 μm square PEDOT:PSS coated Au microelectrodes. The elongated Au traces are connected to the wired external tester. The entire assembly is encapsulated with epoxy resin except for the electrode sites; (**b**) Impedance spectra comparing PEDOT:PSS coated Au and the uncoated Au microelectrodes of the same size.

To verify the electrical characteristics of the post-processed on-chip microelectrodes (Figure 7a), either with Au or combinations of Au and PEDOT:PSS, we performed frequency-dependent impedance measurements in saline (Figure 7b). Our measured impedance values across the frequency range of interest were comparable with the previously reported impedance on conventional planar structures used in electrophysiology, both Au and PEDOT:PSS electrodes [23]. Likewise, we verified that the impedance of the organic PEDOT:PSS was notably lower than that of Au electrodes across the overall frequency range (up to the approximate characteristic frequency of neural action potentials typically used as a reference point for impedance comparison). Here, the impedance of our 70 μm-sized Au electrode (427 kΩ) was more than an order of magnitude larger than that of the 70 μm-sized PEDOT:PSS on Au (7 kΩ) at 1 kHz, consistent with previous findings [23,24]. This result supports that the quality of our post-processing for microfabricated electrodes on the sub-mm sized chip using the lift-off process is comparable with that of electrodes fabricated on the large planar substrate.

### 3.6. Validation of the Post-Processed Microchip Performance as Neural Implants

**Figure 8:**
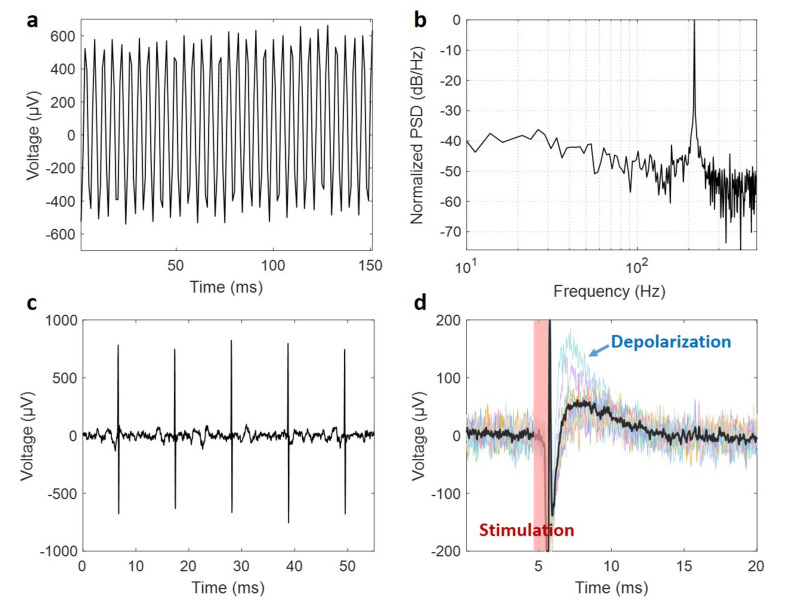
A benchtop saline and ex vivo brain slice experiment validate recording and stimulation microchips functionalized by post-processing: (**a**) a neurograin chip with planar Au electrodes in the recording mode, responding to a 200 Hz sinusoidal current injected into the conductive medium; (**b**) its corresponding power spectral density; (**c**) 100 Hz biphasic stimulation pulses generated in saline by the neurograin chip with PEDOT:PSS electrode; (**d**) The superimposed waveforms of depolarization induced by stimulation current from the chip in a mouse cortical slice.

The performance of the post-processed fully functional “neurograin” CMOS chips has been validated under a benchtop setup in saline and an ex vivo brain slice experiment. We deployed the immersed chips to record wirelessly a 200 Hz sinusoidal “excitation” signal injected into the conductive medium by external microwires. The signal was picked up at the Au electrodes of the wirelessly powered neurograin chips delivered by RF backscattering to external electronics. A raw data telemetry trace is shown in Figure 8a and the corresponding power spectral density (PSD) is in Figure 8b. Additionally, we integrated the hybrid PEDOT:PSS-coated Au electrodes for neurograins operating as wirelessly powered microscale neural stimulators, with the internal circuits designed to generate 100 µs charge-balanced biphasic current pulses (up to 25 µA). Using a 32-channel commercial macroscale multi-electrode array in saline as a wired detector, we measured the neurograin delivered a 100 Hz symmetric stimulation pulse train as shown in Figure 8c. Furthermore, an ex vivo physiological experiment was performed with the wireless stimulation chip array placed onto a mouse cortical brain slice. Figure 8d shows how current injected from one of the wireless microchips into the cortical tissue evoked a large neural circuit response (dominated by large depolarizations), captured by a standard extracellular recording electrode. 

## 4. Conclusions

We proposed a soft material-based, low stress, scalable post-processing method that allowed for conventional microelectronic fabrication techniques on microscale semiconductor chips carrying extremely delicate CMOS integrated circuits (ICs). The proposed post-processing method employed a PDMS embedding approach, widely used in fan-out wafer-level packaging (FOWLP) [40], to allow an additional die-level microelectronic fabrication without damaging the underlying CMOS circuitry. We demonstrated the method on microscale diced and thinned silicon IC chips with lateral dimensions from 280 μm to 650 μm and thicknesses from 30 μm to 200 μm. A PDMS-chip assembly was fabricated into a uniform sheet where CMOS chips are embedded in the soft polymer with a smooth boundary, which results in a spin-coated photoresist to have less than 0.189 μm surface roughness. In addition, by using photoresist-based marker structures, the method allowed precise alignment to form a 2D planarization array. While we have demonstrated this technique in 56 chips, the only limiting factor here is the manual alignment process, which can be automated using a robotic micropositioning system with an imaging tool for a high-speed and high-precision batch fabrication. 

Using this planarization technique, we fabricated both Au and PEDOT:PSS /Au microelectrodes varying size from 15 μm to 200 μm on the contact pads of sub-mm sized CMOS chips through standard photolithography and thin film deposition. Additionally, we introduced a laser ablation as another key step in the post-process to write individual wireless identifiers on multiple chips for their network telecommunication. While validated with a relatively small batch of silicon CMOS chips due to the practical limitation of a semi-automated laser ablation machine, a 2D-planarized chip array formed by the PDMS-based post-processing method would enable us to generate a large number of identifiers on a large batch of wireless IC chips with an automated laser cutting system. Finally, we successfully validated the post-processed microchip performance as neural implants by wireless recording of a sinusoidal signal in saline with a recording neurograin chip and evoking neural activity through current injection from the on-chip planar electrodes of stimulating neurograin chips. 

This technique has been demonstrated in the post-processing of wireless microimplant, which mainly requires single-layer lift-off for electrode patterning. However, other microfabrication processes, such as dry etching, can be combined, as shown in our previous work [41], to make the opening in hermetic packaging. The deformation of PDMS during processing was not observed in this work, even with the use of various chemicals, including photoresists, developers, and stripper. However, some organic solvents can result in swelling of PDMS and thermal decomposition of it can happen at a high temperature above 250 °C, where the thermal stress can already damage CMOS circuits as well.

We anticipate that the proposed post-processing method based on PDMS planarization will offer new opportunities in the development of any miniaturized devices incorporating the materials or structures that are incompatible with a front-end chip fabrication process. Besides our current demonstrations having metal or polymer-metal composite electrodes on foundry-fabricated silicon CMOS chips, sub-mm photovoltaic cells or light-emitting diodes (LEDs) can be handled with the PDMS-assisted technique to attach micropatterned metal or dielectric probes to them in the large-scale batch process, as in the state of the art microLED displays [42]. The proposed method may play a key role in the future semiconductor industry, where the introduction of unconventional materials and structures in conventional IC chips may open up a new opportunity in emerging applications, such as a large network of microscale biomedical implants and environmental sensors.
